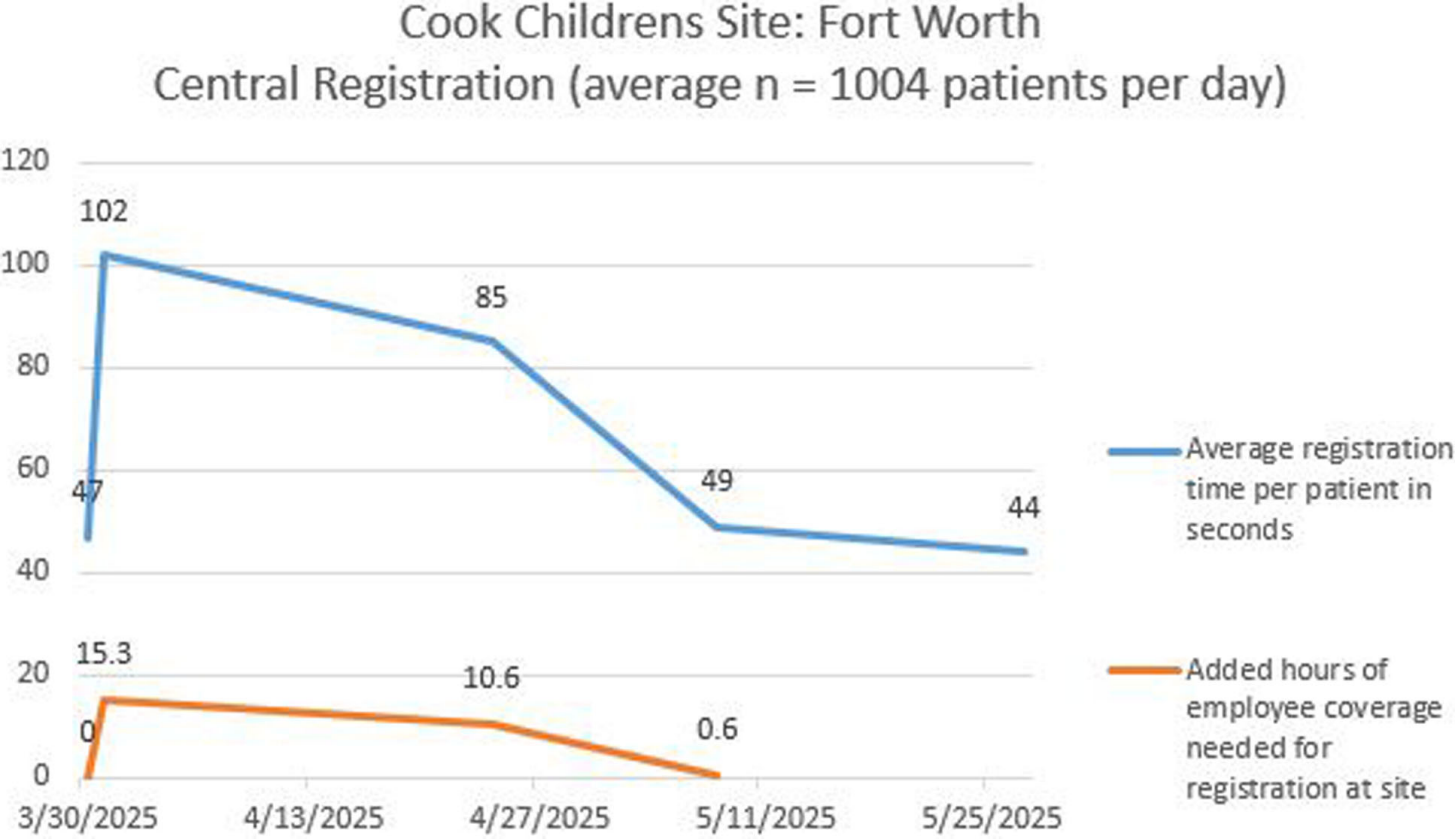# 326 Physician Approach to Indeterminate Clostridioides difficile Test Results

**DOI:** 10.1017/ash.2026.10672

**Published:** 2026-06-23

**Authors:** Marc Mazade, Stacey Rychlik, Cassidy Ware, Yvette Aguire

**Affiliations:** 1 Cooks Childrens Physician Network; 2 Cook Children’s Medical Center

## Abstract

**Background:** Cook Children's has two hospitals in North Texas and a network of clinics extending to the West, where a measles outbreak totalling 762 cases began on January 20, 2025. Measles symptoms include fever, cough, coryza, and conjunctivitis before the onset of the morbilliform rash. Timely isolation is recommended for measles. Isolation should continue from four days before to four days after the onset of rash. We hypothesized that contact, travel, and symptom-based screening could prevent amplification of measles in our healthcare settings and avoid positive screens in most neonates with febrile URI. Administratively, screening is unpopular because screening leads to longer registration times, more manpower requirements, and lower patient satisfaction scores. **Methods:** We selected five measles screening questions employing an adjustable scoring system in which a total score of 5 triggered a positive registration screen, rapid isolation, and further clinician assessment to determine actual testing needs. We scored practice patients by trial and error to settle upon a scheme, then deployed screening at all registration points. We adjusted questions based on evolving local measles epidemiology and redeployed the questions. We monitored throughput. **Results:** Screening for the first iteration started network-wide on March 31, 2025 (Table 1). Scoring included: 1 point for travel to/living in a measles area; 2 points for case exposure; 1 point for no measles vaccination; 2 points for fever; 1 point for ALL 3 C's – cough, congestion, and red eyes. Not anticipating that parents would answer "UNKNOWN" to some questions, a point counted for those instances. Initially, screen positivity was 0.32% (256 of 80,071), while there were 0 PCR-positive cases. The second iteration (55,603 screens) mended the oversight, reducing positive screens to 0.01%. During a third iteration (70,018 screens) following a visitation to a resort by an outbreak-linked case, "ANY," rather than "ALL of the 3 Cs," and/or rash was counted, increasing screen positivity to 0.03%. After a worrisome non-network case presented without travel or known exposure, a fourth iteration (215,957 screens) increased the maximum to 2 points for each 3C or rash (1/2 point for each), so children without travel or known exposure could have a positive screen. Positivity was 0.01%. For iterations 2-4 (341,578 screens), using measles PCR (n=27) positive as the gold standard, sensitivity was 100%, specificity 96%, PPV 50%, & NPV 100% (Table 2). Using "clinician felt PCR was needed" as a gold standard, clinicians ordered PCR in only 5.7% of positive screens and 0.01% of negative screens, including 3 children with fever and vaccine-associated rash only (Table 4). Registration throughput climbed by 117% but returned to baseline by 2 months (Graph). However, managers were pressed into service directing traffic until screening ended July 27, 2025. **Conclusion:** Exposure and symptom-based measles screening can avoid positive screens among low-risk febrile neonates with URI. This model should be employed in other areas to prevent measles transmission before the onset of rash.

**Figure:**
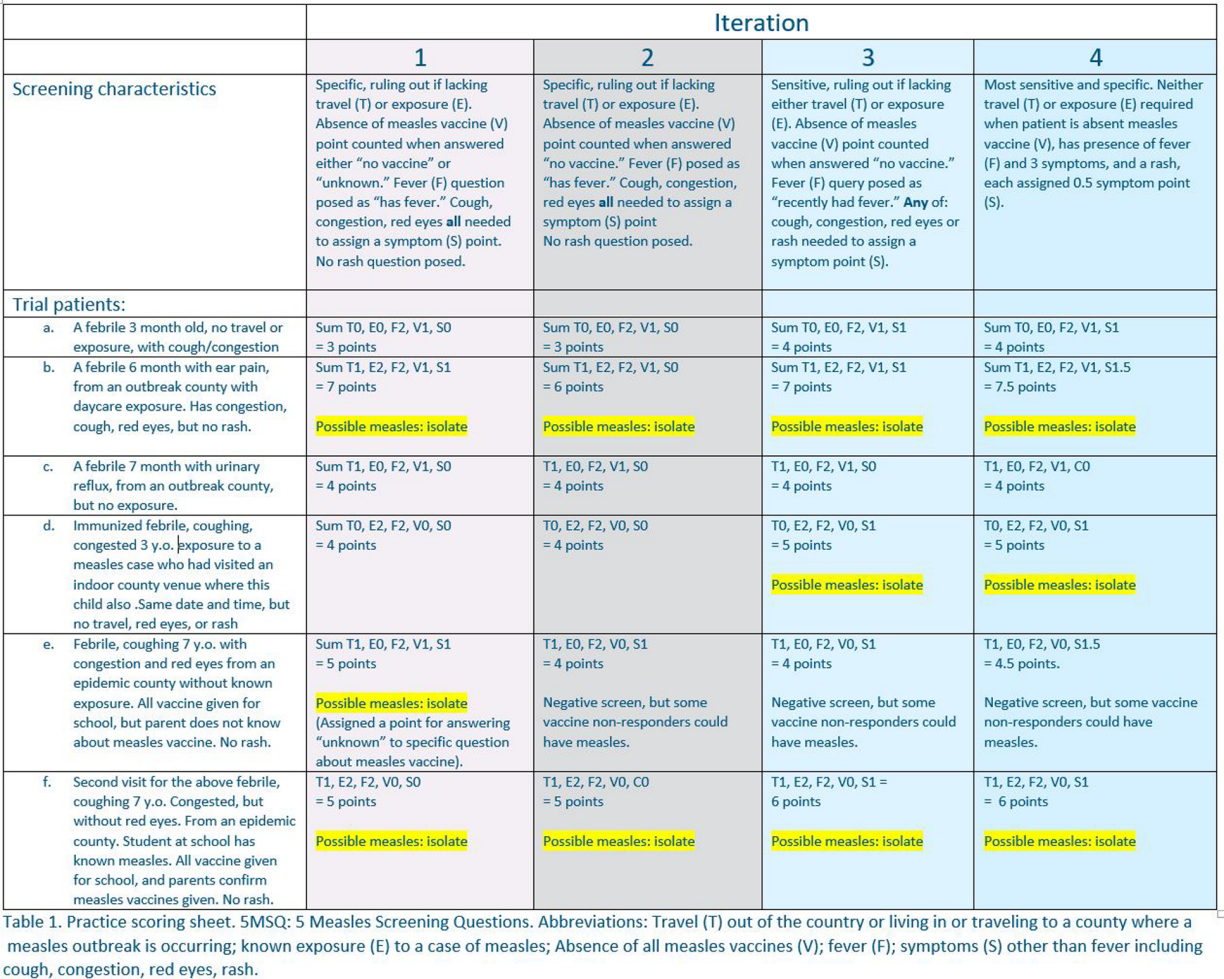

**Figure:**
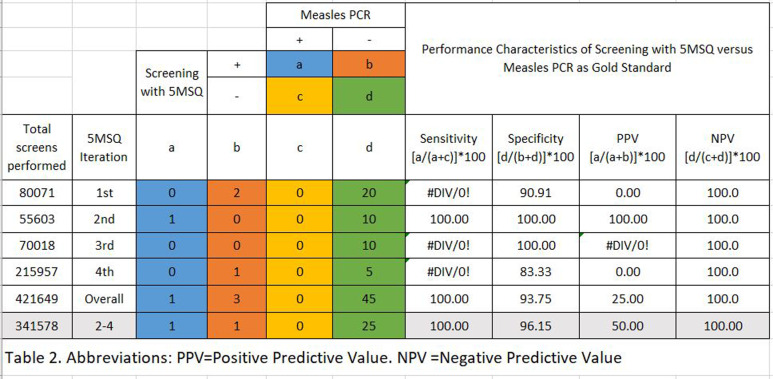

**Figure:**
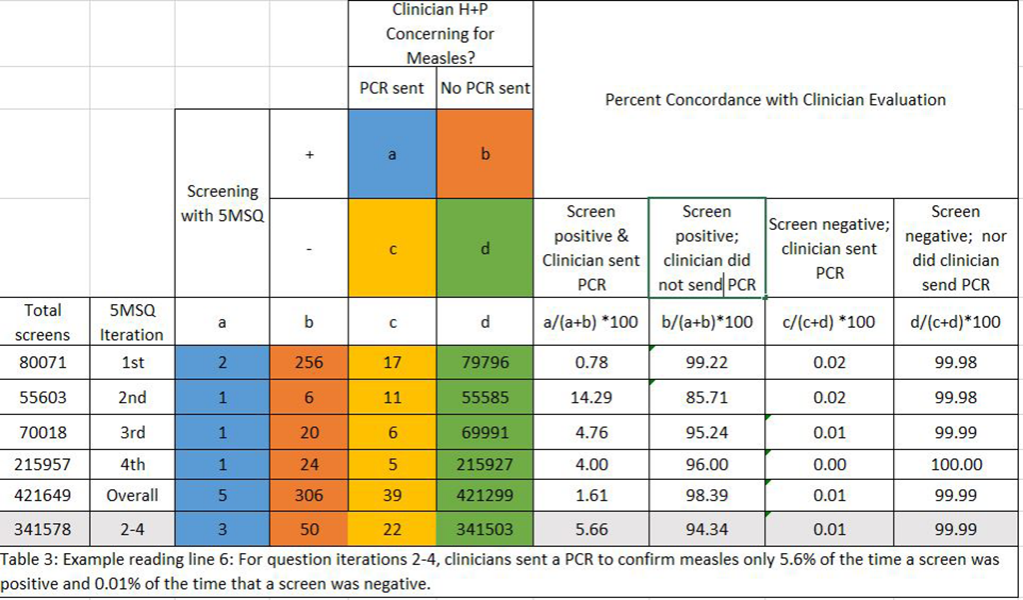

**Figure:**